# Nucleotide-Specific Autoinhibition of Full-Length K-Ras4B Identified by Extensive Conformational Sampling

**DOI:** 10.3389/fmolb.2020.00145

**Published:** 2020-07-10

**Authors:** Balint Dudas, Franci Merzel, Hyunbum Jang, Ruth Nussinov, David Perahia, Erika Balog

**Affiliations:** ^1^Department of Biophysics and Radiation Biology, Semmelweis University, Budapest, Hungary; ^2^Faculty of Information Technology and Bionics, Pázmány Péter Catholic University, Budapest, Hungary; ^3^Theory Department, National Institute of Chemistry, Ljubljana, Slovenia; ^4^Computational Structural Biology Section, Basic Science Program, Frederick National Laboratory for Cancer Research, Frederick, MD, United States; ^5^Department of Human Molecular Genetics and Biochemistry, Sackler School of Medicine, Tel Aviv University, Tel Aviv, Israel; ^6^Laboratoire de Biologie et de Pharmacologie Appliquée, Ecole Normale Supérieure Paris-Saclay, Gif-sur-Yvette, France

**Keywords:** K-Ras4B, KRas4B, autoinhibition, molecular dynamics, normal modes, conformational search, MDeNM

## Abstract

K-Ras is one of the most frequently mutated oncogenes in human tumor cells. It consists of a well-conserved globular catalytic domain and a flexible tail-like hypervariable region (HVR) at its C-terminal end. It plays a key role in signaling networks in proliferation, differentiation, and survival, undergoing a conformational switch between the active and inactive states. It is regulated through the GDP-GTP cycle of the inactive GDP-bound and active GTP-bound states. Here, without imposing any prior constraints, we mapped the interaction pattern between the catalytic domain and the HVR using Molecular Dynamics with excited Normal Modes (MDeNM) starting from an initially extended HVR conformation for both states. Our sampling captured similar interaction patterns in both GDP- and GTP-bound states with shifted populations depending on the bound nucleotide. In the GDP-bound state, the conformations where the HVR interacts with the effector lobe are more populated than in the GTP-bound state, forming a buried thus autoinhibited catalytic site; in the GTP-bound state conformations where the HVR interacts with the allosteric lobe are more populated, overlapping the α3/α4 dimerization interface. The interaction of the GTP with Switch I and Switch II is stronger than that of the GDP in line with a decrease in the fluctuation upon GTP binding.

## Introduction

Members of the Ras (Rat sarcoma) family of small GTPases are conformational switches involved in signal transduction originating from receptor-mediated extracellular signals to the nucleus, controlling cellular proliferation, differentiation, and survival (Hernandez-Alcoceba et al., 2000; Cherfils and Zeghouf, 2013; van Hattum and Waldmann, 2014).

Ras signaling is determined by the GTPase cycle: inactive GDP-bound and active GTP-bound states, which change the conformation of Ras and its affinity to bind to downstream effectors—such as Raf kinase (Pacold et al., 2000; Fetics et al., 2015) and phosphatidylinositol 3-kinase (PI3K) (Pacold et al., 2000). The intrinsically very slow GDP/GTP exchange and GTP hydrolysis rates are increased by two types of regulatory proteins. Guanine nucleotide exchange factors (GEFs) catalyze the release of GDP which is followed by GTP binding, and the GTPase activating proteins (GAPs) catalyze GTP hydrolysis (Bos et al., 2007), resulting in GDP-bound conformations. Oncogenic mutations lock Ras in its active, GTP-bound conformation, being always available to downstream effectors, which leads to uncontrolled cell growth and cancer (Prior et al., 2012). Mutant GDP-bound proteins may also shift their conformations to a GTP-bound-like state.

**Figure 1:**
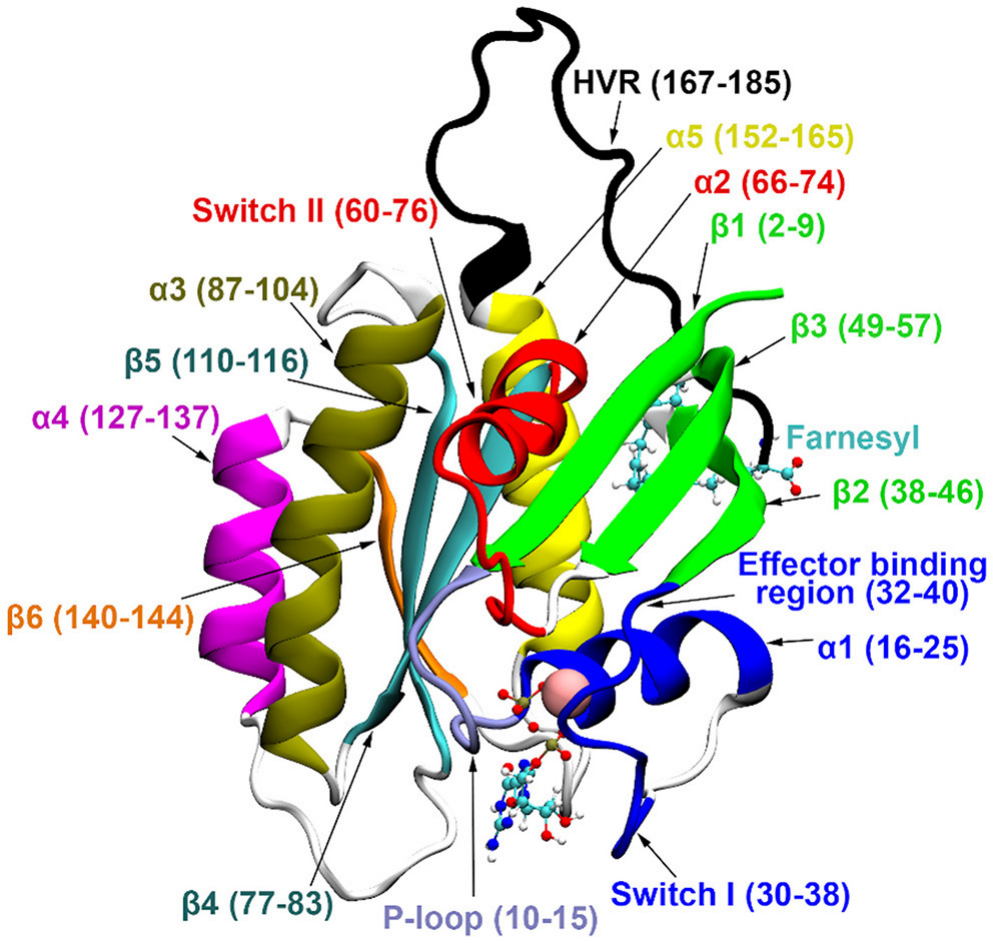
K-Ras4B secondary structure elements. GDP and farnesyl are represented in CPK representation, Mg^2+^ with vdW sphere.

Three main isoforms of human Ras, N-, H-, and K-Ras, the latter having two splice variants, K-Ras4A and K-Ras4B, are known. K-Ras4B has been observed at higher levels (Koera et al., 1997; Plowman et al., 2003) and plays an essential role in cell growth and development. All Ras isoforms share a highly conserved catalytic domain (sequence identity >89%) and a flexible, C-terminal hypervariable region (HVR, sequence homology < 15%) (Castellano and Santos, 2011). For structural details see Figure 1.

To anchor in the membrane and signal, K-Ras4B undergoes post-translational modifications (PTM), including methylation and farnesylation of its CAAX C-terminal box at Cys-185. This farnesyl group together with the lysine-rich HVR direct the interaction of K-Ras4B with the negatively charged lipids of the inner face of the plasma membrane (Brunsveld et al., 2009; Jang et al., 2015, 2016a; Prakash et al., 2016; Prakash and Gorfe, 2017). Emerging nuclear magnetic resonance (NMR) spectroscopy data and computational studies show that in solution, the catalytic domain and the HVR can interact (Abraham et al., 2009; Lu et al., 2015). NMR measurements showed that the HVR of H-Ras dynamically interacts with the catalytic domain, which exhibits increased flexibility in the truncation of the HVR (Thapar et al., 2004). Based on NMR interaction patterns, recent computational studies provided several possible models of the complete K-Ras4B in solution (Chavan et al., 2015; Lu et al., 2015; Jang et al., 2016a). The molecular dynamics (MD) simulations showed that full-length GDP-bound K-Ras4B could promote an autoinhibited state through HVR-catalytic domain interactions, while looser interactions have been detected for the GTP-bound state, which could release autoinhibition. Autoinhibition is typically a transient state, which explains the difficulties in obtaining its structure experimentally, e.g., by NMR or crystallography. Autoinhibition protects against spurious activation and proteolysis (reviewed in Nussinov et al., 2018). Even if the interaction of the autoinhibiting segment is weak, its large population at the active/functional site effectively shields it, which explains why oncogenic drivers often aim to release it (Nussinov et al., 2020).

Here, we perform a conformational space mapping of the full-length GDP- and GTP-bound K-Ras4B in solution without imposing any prior knowledge or constraints on the catalytic domain-HVR interactions. We start from an extended HVR conformation and use a computational method that combines MD and the normal mode approach, called Molecular Dynamics with excited Normal Modes (MDeNM) (Costa et al., 2015). In agreement with previous studies, we found that GDP-bound full-length K-Ras4B favors an autoinhibited conformation, while in solution the GTP-bound protein favors a conformation where the autoinhibition is lifted leading to an active state. The autoinhibition in the GDP-bound state is realized by the interaction of HVR with Switch I and Switch II at the effector lobe, thereby blocking the effector binding site. In turn, the conformations populated in the GTP-bound state exhibit an HVR interaction with α4 at the allosteric lobe, thus blocking the α3/α4 dimerization site. In the GDP-bound state, these conformations hardly exist. In particular, our results show that compared to the GDP-bound form, the interaction of the GTP nucleotide is stronger with both Switch I and Switch II in agreement with the decrease in fluctuation upon GTP binding, and the measured binding affinity, Kd values (John et al., 1993).

## Materials and Methods

Molecular Dynamics with excited Normal Modes (MDeNM) (Costa et al., 2015) simulations were carried out on full-length (residues 1-185) farnesylated and methylated (FME) K-Ras4B in its inactive GDP-bound and active GTP-bound forms. The starting coordinates of the catalytic domain were taken from crystal structures with PDB ID 4OBE (Hunter et al., 2014) and 3GFT, respectively. For the active state, the GTP analog GppNHp was modified to GTP, and His61 was mutated back to the native Gln61.

MDeNM simulations and analysis were performed with CHARMM (Brooks et al., 2009) using CHARMM all-atom additive force field C36 (Best et al., 2012) while conventional MD simulations were carried out with NAMD (Phillips et al., 2005) using the same CHARMM force field mentioned above. The GDP/GTP parameters were combined from the ADP and guanine parameters existing in CHARMM, while the parameters for farnesylated Cys were taken from our previous studies (Jang et al., 2016b).

Our main objective in carrying out the MDeNM simulations is a deep exploration of the conformational preferences of the two Ras forms. The structures of the catalytic domain deposited in Protein Data Bank differ in length (e.g., in 4EPT the structure is determined up to amino acid 166; in 3GFT up to 167, in 4OBE to 169), indicating that the C-terminal amino acids have higher fluctuation. Consequently, for the GDP bound state we created three initial structures with different HVR orientations. In these structures, the HVR was built starting at residues 167, 168, and 169, respectively, from the internal coordinate table of CHARMM, yielding a linear conformation distant from the catalytic domain. In order to relax the HVR, the first 100 steps of steepest descent were followed by 1,000 steps of adopted basis Newton-Raphson energy minimization. Then the HVR of these structures was heated to 300 K in 10 ps followed by a 90 ps equilibration while the catalytic domain was kept fixed. This procedure leads to HVR conformations that face different sides of the catalytic domain, as can be seen in Supplementary Figure 1. In order to have the same initial HVR conformations to study the nucleotide dependent catalytic domain-HVR interactions in an unbiased manner, the same three orientations of the HVR were applied to the GTP-bound state using 3GFT for the structure of the catalytic domain as described above.

The six obtained structures were solvated using CHARMM-GUI (Jo et al., 2008; Lee et al., 2016). In all cases, a rectangular box containing TIP3 water molecules extending 15 Å in all directions from the surface of the protein was generated with a concentration of 0.10 M NaCl. For the energy calculations, the dielectric constant was set to 1. The Particle Mesh Ewald (PME) method was used to calculate the electrostatic interactions with a grid spacing of 1 Å or less having the order of 6; the real space summation was truncated at 12.0 Å, and the width of the Gaussian distribution was set to 0.34 Å^−1^. Van der Waals (vdW) interactions were reduced to zero by “switch” truncation operating between 10.0 and 12.0 Å.

Solvated systems were energy minimized with progressively decreasing harmonic restraints: first, the steepest descent was used with the harmonic force constant decreased every 500 steps of 10, 1, and 0.1 kcal/mol/Å^2^, followed by 200 steps of conjugate gradient with a 0.1 kcal/mol/Å^2^ force constant. Unrestrained minimization was then applied for 100 steps with the steepest descent, 200 steps with the conjugate gradient, and 1,000 steps with the adopted basis Newton-Raphson method. The energy-minimized structures were heated and equilibrated at 300 K for 200 ps in an NVT ensemble, followed by a 5 ns NPT run at a pressure of 1 atm. The Langevin dynamics was used with the damping coefficient of 1 ps^−1^, a piston oscillation period of 50 fs, and a piston oscillation decay time of 25 fs. The integration time step was set to 2 fs.

### MDeNM Simulations

In order to map the conformational space more efficiently than with classical MD simulations, the Molecular Dynamics with excited Normal Modes (MDeNM) (Costa et al., 2015) method was used. The normal modes necessary for the MDeNM simulations were calculated by considering the final structures resulting from the 5 ns MD run for both GDP- and GTP-bound FME full-length K-Ras4B. The energy of the two structures was minimized using the steepest descent method, the harmonic force constant decreasing every 1,000 steps, adopting the values 10, 1, 0.1, and 0 kcal/mol/Å^2^, followed by 50,000 steps of adopted basis Newton-Raphson method. After the energy minimization, the normal modes were calculated using the VIBRAN module of CHARMM. For the MDeNM calculations, based on their RMSF contribution, the 10 lowest frequency normal modes were taken.

In the second step, the final structure of the 5 ns MD run for all 3 models of both GDP- and GTP-bound states (in total six systems) was considered as initial structures for MDeNM simulations: randomized linear combinations of the first 10 lowest frequency normal modes were generated, giving excitation directions. These excitation directions were then used to kinetically excite the systems during MD simulations yielding to different replicas. The excitations were carried out during the MD simulations with successive kinetic energy injection along the direction of the combined mode in the form of velocity increment equivalent to an overall 10 K temperature increment of the system. Excitations were performed in the same direction at every 2,500 steps of the MD simulation, giving a relaxation time of 5 ps for the system in each excitation cycle. The dissipation of the energy inserted in each cycle was checked by to ensure that there is no appreciable accumulation of kinetic energy along the excitation direction. Each period of excitation-relaxation yields a given conformation. The other parameters of the MD remained the same as those described above.

In total 264 MDeNM replica simulations were carried out corresponding to different NM combination directions for the three models in GDP- and GTP-bound states. In order to ensure an exhaustive search of the conformational space, the newly generated replicas were compared to the previously accepted ones and were only kept if the root-mean-square deviation (RMSD) value—between the structures displaced by 1 Å along the mode combinations—was greater than 1.65 Å. 32 excitations per replica were generated, resulting in 8,448 structures for each of the systems.

### MD Simulations

In order to compare the conformational space mapped by MDeNM to the conformations accessible by conventional MD simulation, three parallel 200 ns long MD simulations were performed for both the GDP- and GTP-bound states, having the same starting structures as the MDeNM simulations (i.e., the final structure of the 5 ns equilibration run). The parameters for the 200 ns run were identical to those of the 5 ns equilibration.

### Interaction Energy Calculations

The binding preferences of the HVR with the allosteric or effector lobe regions in the GDP- or GTP-bound K-Ras4B were analyzed by comparing distributions of HVR interaction energies, Δ*E*_*HVR*_. The interaction energy of a given structure was evaluated as a difference between the energies of bound and a reference unbound state in which the HVR is completely detached, as given by

(1) $$\begin{array}{r} \Delta E_{\text{HVR}\left(\text{a}/\text{e}\right)}=E_{\text{bound}\left(\text{a}/\text{e}\right)}-\langle E_{\text{non}-\text{bound}}\rangle \;\left(1\right) \end{array}$$

where *E*_*bound*(*a*/*e*)_ is the HVR interaction energy of a structure within either the allosteric (a) or effector (e) lobe and <*E*_*non*−*bound*_> the reference energy of its unbound state. The interaction energy is evaluated as a sum of pairwise electrostatic and vdW potential energy contributions between the HVR and its environment including the protein and the solvent by using CHARMM36 force field. The interaction energy of the reference state, in which the HVR is exposed exclusively to the solvent composed of explicit water molecules and ions, is obtained as an average over a sufficiently large number of conformations (15,500) to ensure a converging value. The simulations were carried out using the Hungarian KIFU supercomputing facility.

## Results and Discussion

### Interactions of the HVR With the Catalytic Domain

In order to identify the interacting residues of the catalytic domain and the HVR, a distance-based criterion was applied to the conformations generated by MDeNM: if the distance between two heavy atoms of a residue within the catalytic domain and a residue in the HVR was less than 5.5 Å, these two residues were considered interacting (Bowerman and Wereszczynski, 2016). Based on this criterion, our analysis shows that the interacting catalytic domain-HVR residues detected by MDeNM contain all the interacting residues identified by NMR measurements (Thapar et al., 2004; Chavan et al., 2015; Lu et al., 2015; Jang et al., 2016a).

**Figure 2:**
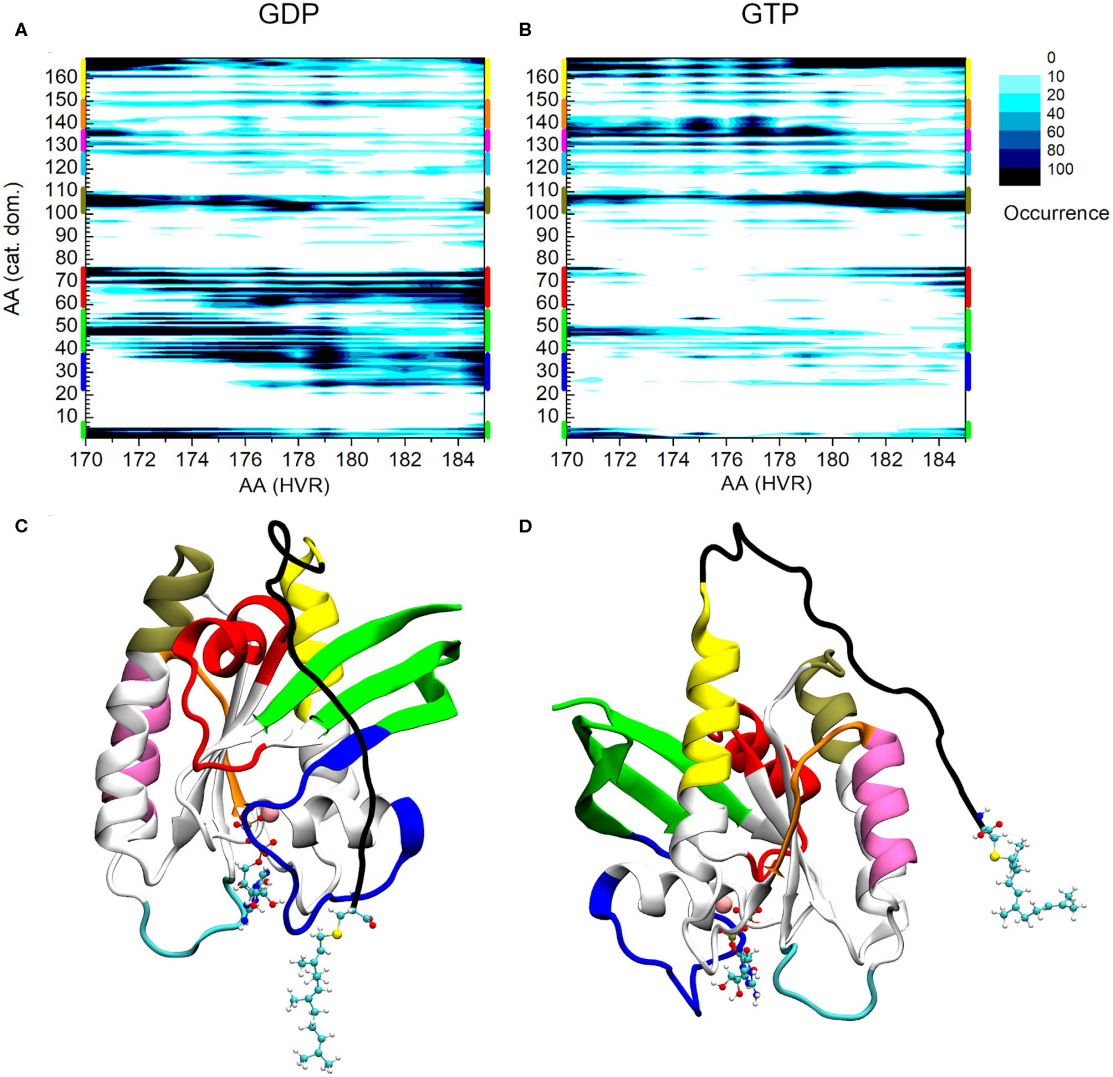
Distance-based interaction map between the catalytic domain and the HVR of K-Ras4B. **(A)** GDP-bound, **(B)** GTP-bound state. **(C)** 3D structure of K-Ras4B with the effector lobe and **(D):** the allosteric lobe in front. The nucleotide and the farnesyl group are represented as CPK, Mg^2+^ as VDW sphere. Structural regions of the catalytic domain color-coded along the y-axis of **(A)** and **(B)** are mapped with identical coloring onto the C and D part of the figure.

Figure 2 shows the interaction map based on the calculations described above for both the GDP- and GTP-bound structures. The number of interactions within a given population goes from white (no structure with such an interaction) to black (maximum number of structures with the given interaction), as indicated in the legend. On the x-axis HVR residue numbers, on the y-axis those of the catalytic domain are presented. Figure 2A shows the pairs of interacting residues for the GDP-bound state, Figure 2B those for the GTP-bound state. In order to identify the structural elements of the catalytic domain which interact with the HVR, different regions are designated by bars of different colors on the vertical edges of the graphs. The same color coding is kept for Figures 2C,D, where the three-dimensional structure of the protein is represented from different views.

A striking difference can be observed between the GDP- and GTP-bound states. In the GDP-bound form, the HVR shows extensive interactions with Switch I (blue) (including the effector binding site), β1, β2, and β3 (green), and Switch II (red) (Figure 2A), while in the GTP-bound form, these interactions are either sparsely populated or non-existent (Figure 2B). This is in agreement with previous observations (Chavan et al., 2015; Lu et al., 2015; Jang et al., 2016a), demonstrating that in the GDP-bound state, the HVR hinder approach to the effector binding site, by overlapping with Switch I and Switch II, brings the system to autoinhibition (Figure 2C).

For the GTP-bound state, there are interactions between the very first residues (170-173) of the HVR and the N-terminal part of the catalytic domain (residues 1-6) and the loop, L3, (residues 47-50)—which connects β1 and β2 strands (noted in green) (Figure 2B). However, the interactions between the second part of the HVR and Switch I (blue) and Switch II (red) are sparsely populated with only the last residues at the farnesylated C-terminal end of the HVR interacting with this region. This indicates that in the GTP-bound state, the C-terminal end of the HVR shifts toward the allosteric lobe or is detached from the catalytic domain (Figure 2D), but the interaction with the effector lobe is very weakly populated.

The olive-colored region shows the HVR interaction with the C-terminal end of α3 and the loop between α3-β5, while the yellow-colored region shows the HVR interaction with the C-terminal end of α5 (Figures 2C,D). The main difference between the GDP- and GTP-bound states is that in the GDP-bound form, the N-terminal region of the HVR interacts with α3/α5, and the C-terminal region interacts with the Switch II region at the effector lobe. However, in the GTP-bound form, the more populated interactions extend to the C-terminal region of the HVR that interacts with the catalytic domain residues located in spatial proximity of the N-terminal region of HVR, such HVR forming a loop by itself.

Unlike the interacting regions in the effector lobe, HVR interactions at the regions designated by purple and orange of the allosteric lobe are almost non-existent in the GDP-bound state, while they are strongly present in the GTP-bound form. We observed that HVR residues, Asp173, Lys175, and Lys177 interact with residues 135-142 of α4 (noted purple), loop L9 and β6 (noted orange) in the catalytic domain. Interestingly, this region is part of the allosteric Ca^2+^-acetate binding site described by (Buhrman et al., 2010).

A possible dimerization interface of K-Ras4B was observed at the α3/α4 region of the allosteric lobe (Muratcioglu et al., 2015). In this dimerization mode, the effector binding sites are exposed, allowing the recruitment of Raf and its dimerization, which is a prerequisite for its activation (Inouye et al., 2000). Our results indicate that the HVR-α4 interaction, which is almost non-existent in the GDP-bound form, but highly populated in the GTP-bound state, disfavors dimerization at this helical interface in the GTP-bound form in solution. On the other hand, in the GDP-bound state with the HVR associating with the effector binding region, it blocks the β-sheet dimer formation interface (Muratcioglu et al., 2015). These results may explain why GTP-bound K-Ras4B is predominantly monomeric in solution, even at high protein concentrations. *In vivo*, in the presence of the membrane, the HVR largely associates with the membrane in the GTP-bound state, so this GTP-bound inhibitory scenario is unlikely to play a significant role. Nonetheless, it still provides some insight into the inherent tendencies of the HVR behavior.

To understand the differences in the sparsity of the distance map between GDP- and GTP-bound states, we calculated the interaction energy between the HVR and the catalytic domain, Δ*E*_*HVR*_. The conformations generated by the simulation protocol (above) were divided into three groups: (i) conformations with the HVR detached from the catalytic domain giving rise to the reference state, (ii) conformations with the HVR interacting with the effector lobe of the catalytic domain, and (iii) conformations with the HVR interacting with the allosteric lobe of the catalytic domain. Interaction energies of the HVR were then calculated in the presence of explicit solvent for all conformations in the three groups according to Equation (1).

**Figure 3:**
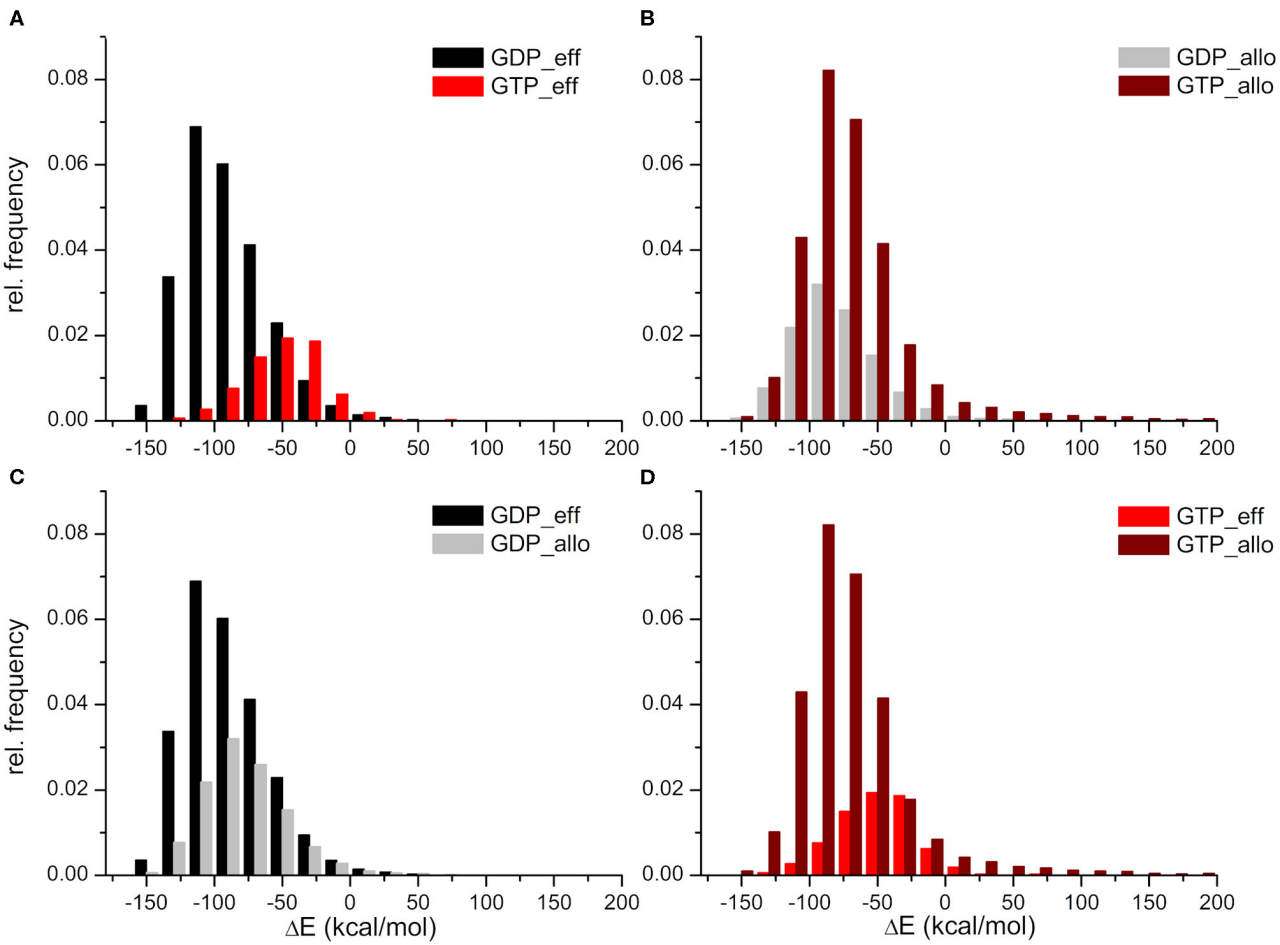
Distributions of interaction energies between the HVR and the catalytic domain of K-Ras4B, within the GDP- and GTP-bound population. HVR interactions **(A)** with the effector lobe in the GDP-bound (black) and GTP-bound (red) state; **(B)** with the allosteric lobe in the GDP-bound (gray) and GTP-bound (wine) state; **(C)** with the effector (black) and the allosteric (gray) lobe in the GDP-bound state; and finally **(D)** with the effector (red) and the allosteric (wine) lobe in the GTP-bound state.

The HVR interaction energy distributions illustrate a higher relative frequency of structures when the HVR interacts with the effector lobe within the GDP- than the GTP-bound population with respect to their whole structural population (Figure 3A). In contrast, higher relative frequency can be seen of the HVR interaction with the allosteric lobe in the GTP-bound state (Figure 3B). This suggests that the HVR of GDP-bound K-Ras4B favors the interaction with the effector lobe (Figure 3C), while that of GTP-bound K-Ras4B tends to reside in the allosteric lobe (Figure 3D), resulting in the differences in the sparsity between the lower parts of the distance based interaction maps (Figures 2A,B). Figure 3A also shows that the GDP-bound interaction energy distribution falls deeper, indicating stronger interactions of the HVR with the effector lobe within the GDP-bound than the GTP-bound population. While (as Figure 3B shows) in the HVR interaction with the allosteric lobe, we observe that for both GDP- and GTP-bound states the peaks of the distributions are located close to each other, indicating no difference in interaction energy of the HVR with the allosteric lobe irrespectively of the bound nucleotide.

In summary, whereas in the GDP-bound state interactions with the effector lobe are more populated and are shifted toward the low energy values, in the GTP-bound state, it is the opposite: structures interacting with the allosteric lobe are much more numerous than those interacting with the effector lobe, and the interaction energy of HVR with the allosteric lobe falls deeper than with the effector lobe.

### Interactions of the Nucleotides With the Switch Regions

**Figure 4:**
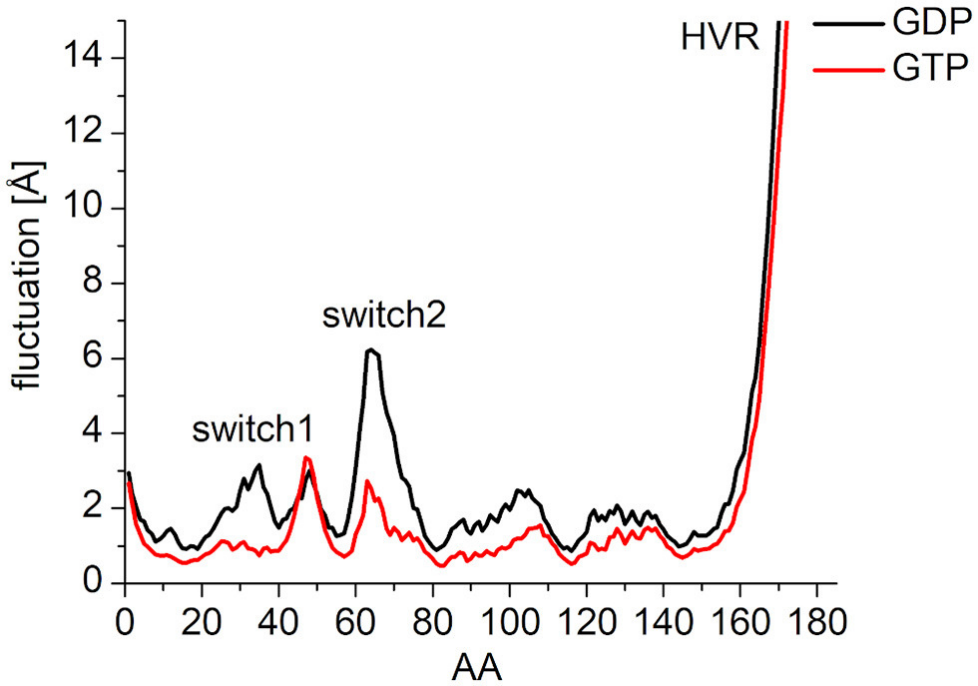
RMS fluctuation of C_α_ atoms on MDeNM generated conformations of GDP- (black) and GTP-bound (red) K-Ras4B. For the better visualization of the catalytic domain regions the y-axis is enlarged up to and cropped at 15 Å.

In order to characterize the interaction of the nucleotides with the catalytic site, the root-mean-square fluctuation (RMSF) of the C_α_ atoms on the MDeNM generated conformations for both GDP- and GTP-bound forms was calculated (Figure 4). This agrees with the previous findings: in the calculations, we observed that the GDP-bound form exhibits a higher RMSF at both Switch I and Switch II regions. As expected, the HVR (residues 167-185) has a considerably larger fluctuation in both systems compared to the catalytic domain.

**Figure 5:**
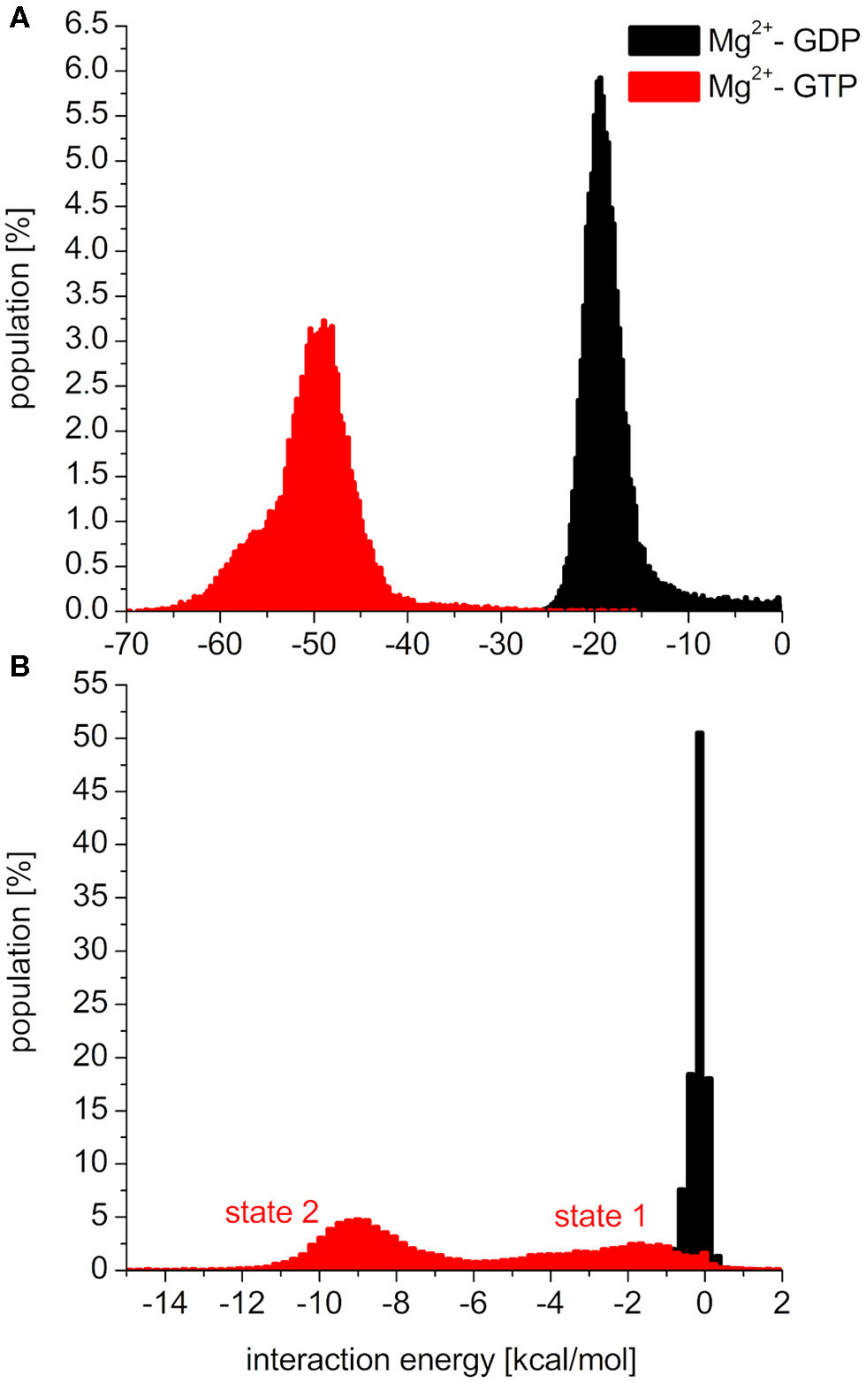
Distributions of interaction energies between the nucleotide (Mg^2+^-GDP: black, Mg^2+^-GTP: red) and **(A)**: switch1 region, **(B)**: switch2 region of K-Ras4B.

To elucidate the behavior of the switch regions from an energetic point of view, the interaction energies between the nucleotides (with the coordinating Mg^2+^: Mg^2+^-GDP/Mg^2+^-GTP) and the Switch I/Switch II regions were calculated for the MDeNM conformations. In the interaction with Switch I (Figure 5A), the interaction energy of Mg^2+^-GTP is considerably more favorable than that of Mg^2+^-GDP, suggesting the well-known stiffening conformation of Switch I in the active form, which is more available to downstream effectors (Cherfils and Zeghouf, 2013; Lu et al., 2016). In the interaction with Switch II (Figure 5B), the Mg^2+^-GTP-bound population shows two peaks in its distribution, corresponding to two different conformational states, both falling deeper than that of the Mg^2+^-GDP, which is in agreement with the decrease of RMSF in the GTP-bound form and explains the stronger binding of GTP vs. GDP (John et al., 1993).

**Figure 6:**
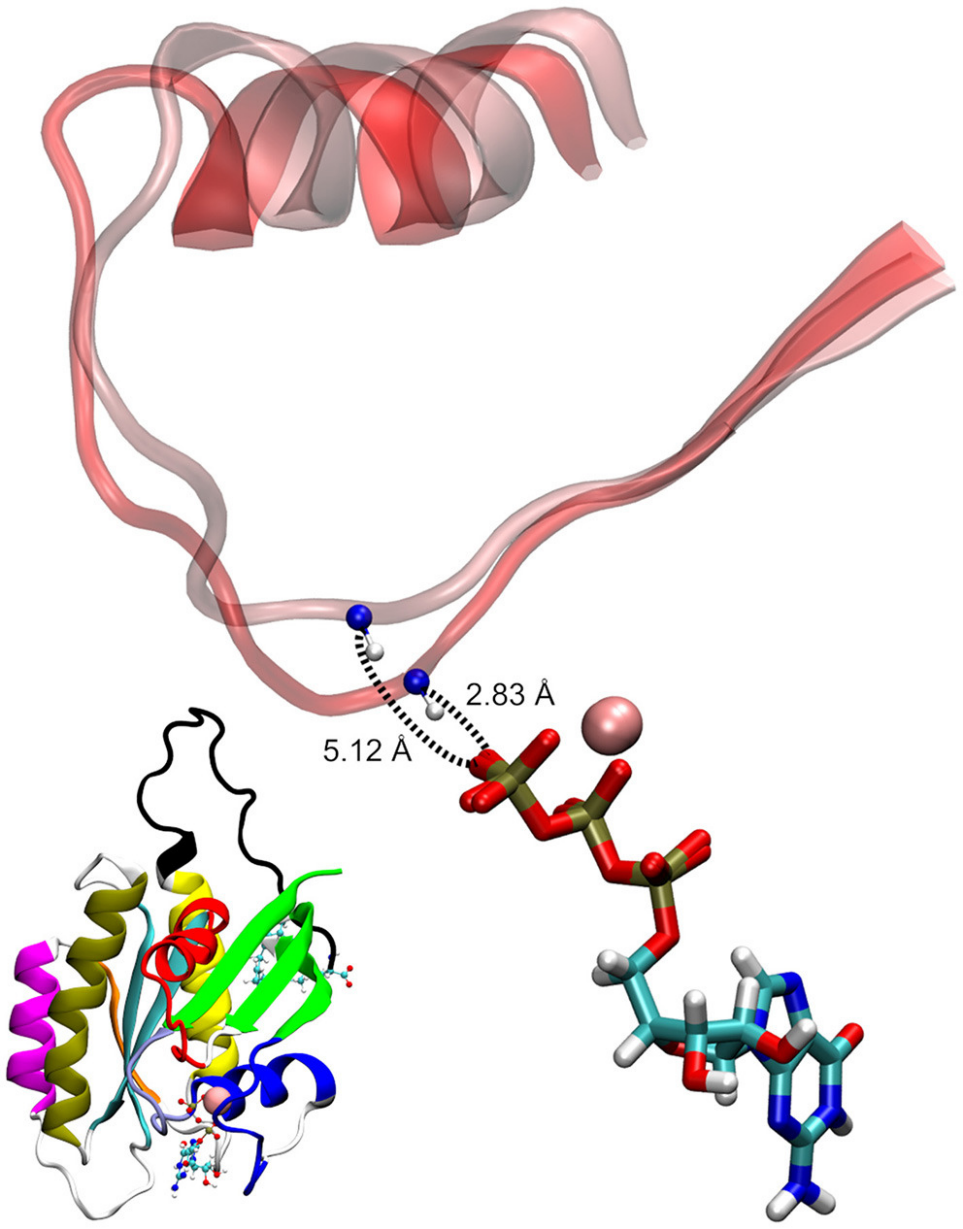
Examples of GTP-bound K-Ras4B in state 1 and state 2, superimposed at their nucleotides. The shorter donor-acceptor distance corresponds to state 2.

As seen in Figure 5B, the two conformational states of Mg^2+^-GTP/Switch II are likely to be identical to state 1 (higher energy conformation) and to state 2 (lower energy conformation) (Spoerner et al., 2010), which are in chemical equilibrium in solution. State 1 was identified as having a low affinity for effectors and low intrinsic hydrolysis, and state 2 as an “effector-binding state” showing high affinity for effectors and being stabilized by them. Figure 6 shows the environment of a conformation from state 1 and from state 2 with superimposed nucleotides. The conformations of state 1 show weaker interactions between the nucleotide (Mg^2+^-GTP) and the Switch II, lacking the H-bond formed between the donor N atom of Gly60 and the oxygen acceptor atom of γ-phosphate of GTP. This H-bond only exists in the state 2 conformations. Since this strong H-bond is present in the initial structure, showing a distance of 2.80 Å between GTP:O1G (acceptor) - Gly60:N (donor), the existence of the state 1 can be interpreted as the result of the moderate excitation kinetic energies introduced by MDeNM being capable of breaking the H-bond and contributing to a more fluctuating Switch II, resembling the inactive, GDP-bound K-Ras4B. This interaction is completely absent due to the lack of γ-phosphate in the GDP-bound state.

As stated previously, the HVR has a considerably larger fluctuation in both systems compared to the catalytic domain. To elucidate the internal flexibility of the HVR, we analyzed the rotational angle space of its residues. The dihedral angles defined by consecutive Cα atom quadruplets were calculated within the HVR for the GDP- and GTP-bound population (Supplementary Figure 2). The base of the HVR (N-terminal part of it) shows a bent configuration in both states with a richer rotational distribution in the case of the GDP-bound state. Both states exhibit a distributional peak corresponding to a rather elongated configuration for the parts following the bend of the base of the HVR. The difference between the activation states is that the HVR of the GTP-bound population has a slightly more restricted rotational profile; still, in some regions, two population peaks are observed one corresponding to an elongated structure and the other to a sharp bending located at the residue levels of 169, 172, and 175.

### Comparison of MDeNM and MD

**Figure 7:**
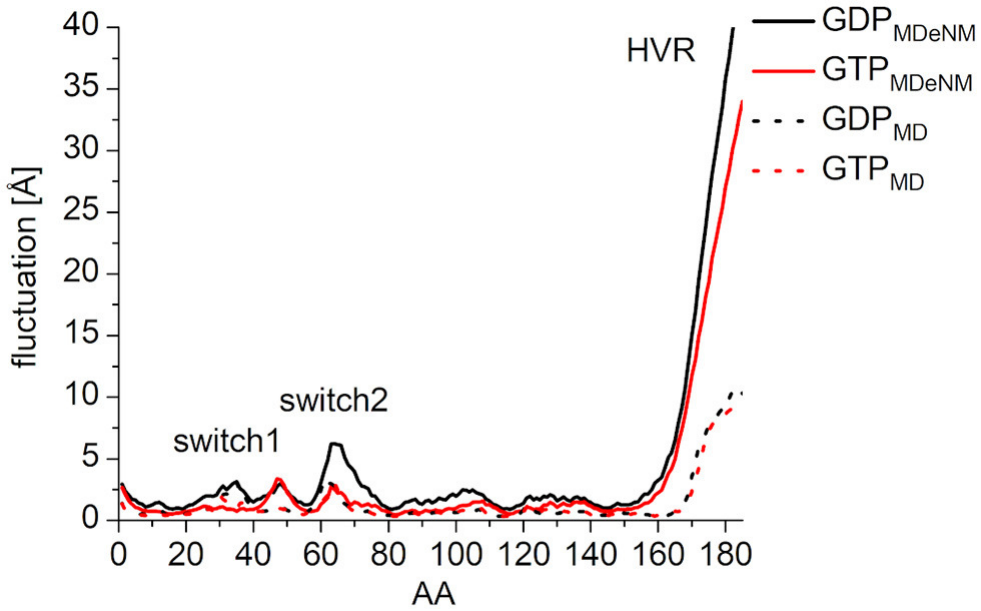
RMS fluctuation of C_α_ atoms among the MDeNM (solid) and MD (dotted) generated conformations for GDP- (black) and GTP-bound (red) K-Ras4B.

In order to compare the conformational space mapped by MDeNM to conformations accessible by classical MD simulation, three parallel MD simulations of 200 ns were performed for both the GDP- and GTP-bound forms, having the same starting structures as the MDeNM simulations. In general, the RMSF follows the same pattern both on MDeNM (solid line) and MD (dotted line) generated structures (Figure 7). However, two major differences can be noted: (i) the HVR shows approximately four times higher values for MDeNM compared to the MD conformations, demonstrating that MDeNM maps a considerably larger HVR conformational space; (ii) the GDP-bound Switch II shows a 2-fold RMSF in the case of MDeNM compared to the classical MD. This difference is not visible in the GTP-bound form, due to the presence of γ-phosphate.

**Figure 8:**
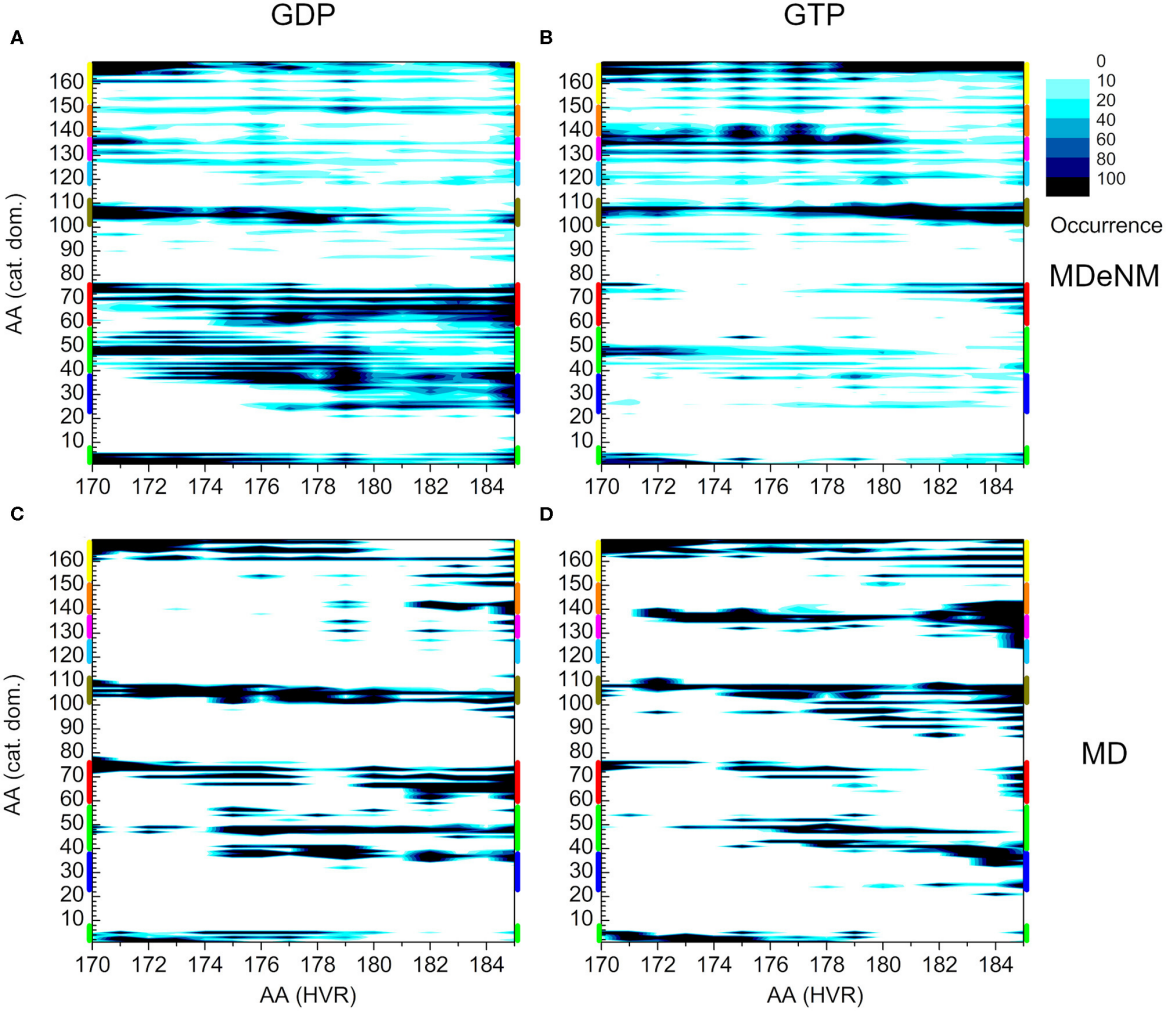
Distance-based interaction map between the catalytic domain and the HVR of K-Ras4B. **(A)** and **(B)** correspond to conformations of MDeNM for the GDP- and GTP-bound population, respectively. **(C)** and **(D)** correspond to conformations of MD for the GDP- and GTP-bound state, respectively. The color-coding of the structural regions within the catalytic domain along the y-axis is identical to Figure 2.

To compare the two methods, we present the distance-based interaction maps between the catalytic domain and HVR among the conformations of both MDeNM (Figures 8A,B) and MD (Figures 8C,D) simulations for the two nucleotide-binding states. The striking difference between the results of the two methods is that the HVR-catalytic domain contacts are more dispersed in the case of MDeNM than for classical MD, the latter being more localized. For the GDP-bound form (Figures 8A,C), both methods show contacts between the HVR and the C-terminal of the catalytic domain (yellow); the C-terminal end of α3 (olive); α2 (red); β1, β2, β3 (green); the C-terminal end of α4 (magenta) and β6 (orange), which simply form the “northern hemisphere” of the catalytic domain, with the north-pole being the N-terminal region of HVR. The major difference between MDeNM and MD manifested at the active site of the protein is the degree of autoinhibition, which is more populated by MDeNM and the extent of the HVR-Switch I/Switch II (blue and red respectively) contact and its versatility being greater again by MDeNM. The GTP-bound form also shows contact with the “northern hemisphere” (yellow, olive, red, green, magenta, orange) indicating a broader conformational mapping with the MDeNM method than with the classical MD. Two regions are more populated in the case of MD than MDeNM: the interaction of the farnesylated C-terminal end with α4/β6 (magenta/orange), and with Switch I (blue). In both cases, visual examination of the MD trajectory revealed that after the HVR found an energetically favorable position it spends considerably more time around the given local minima before moving further, thus exploring the conformational space in a more limited manner.

Another measure to compare the ensembles generated by MDeNM and MD for both states of the enzyme is the overall distribution of the RMSD values with respect to the starting structure (Supplementary Figure 3). If the RMSD of the catalytic domain (without HVR) is calculated, the distribution of the RMSD for the GDP- bound state is 1.9 ± 1.7 Å on the MDeNM, and 1.5 ± 1.2 Å on the MD generated ensemble, while for the GTP-bound state it is 1.7 ± 1.5 Å for MDeNM and 1.2 ± 0.2 Å for MD. On one hand, these values demonstrate that the GDP-bound catalytic domain is more flexible than the GTP-bound one; on the other hand, they also show that MDeNM explores a larger conformational space (both the average RMSD values and the deviations are higher).

If for the RMSD calculations the full-length protein is considered, for the GDP-bound state the values are 12.1 ± 4.0 Å for MDeNM and 11.6 ± 1.9 Å for MD, while for the GTP-bound state 12.4 ± 4.0 Å on the MDeNM and 10.8 ± 2.1 Å on the MD generated structures. The tendency in the full-length case is similar to the one shown for the catalytic domain by itself, magnified by the fluctuation of the HVR.

## Conclusion

As MDeNM revealed and was confirmed by MD simulations, almost all catalytic domain-HVR interactions exist in both GDP- and GTP-bound K-Ras4B. In the GDP-bound form, the population of the conformers is shifted toward a state where the farnesylated C-terminal HVR interacts with the effector lobe of the catalytic domain, blocking both effector binding and dimerization through β-sheet formation. In contrast, in the GTP-bound form, these conformations either do not exist or are poorly populated. In addition, in the GTP-bound state, the population of the conformers is shifted in such a manner that becomes highly populated when the HVR interacts with the allosteric lobe, exposing the effector binding sites. The GTP-bound form provides the HVR conformation overlapping with the α-helix dimer interface at the allosteric lobe, but these conformations are much less populated in the GDP-bound state.

Thus, MDeNM proves capable of an extensive sampling of the interaction of the HVR with the catalytic domain, capturing ensembles that were shown earlier for the GDP-bound K-Ras4B states and revealing new distributions for the GTP-bound states where HVR binding overlaps the α3/α4 dimerization surface. Even though in the presence of the membrane the extent of these interactions is unclear, MDeNM captures the HVR tendencies, which may nonetheless be partially populated.

## Data Availability Statement

The raw data supporting the conclusions of this article will be made available by the authors, without undue reservation, to any qualified researcher.

## Author Contributions

This work was designed by EB, DP, and RN. The simulations were carried out by BD, and were analyzed by BD and FM. The manuscript was written by BD, FM, HJ, RN, DP, and EB. All authors contributed to the article and approved the submitted version.

## Conflict of Interest

The authors declare that the research was conducted in the absence of any commercial or financial relationships that could be construed as a potential conflict of interest. The handling editor declared a past collaboration with one of the authors DP.
