# Supplementary material for: Nucleotide-Specific Autoinhibition of Full-Length K-Ras4B Identified by Extensive Conformational Sampling
Source: Front Mol Biosci. 2020 Jul 10;7:145. doi: 10.3389/fmolb.2020.00145 (PMC7366858; doi:10.3389/fmolb.2020.00145)
Supplement: Supplementary file 1 [file Table_1.DOCX]

Supplementary Material


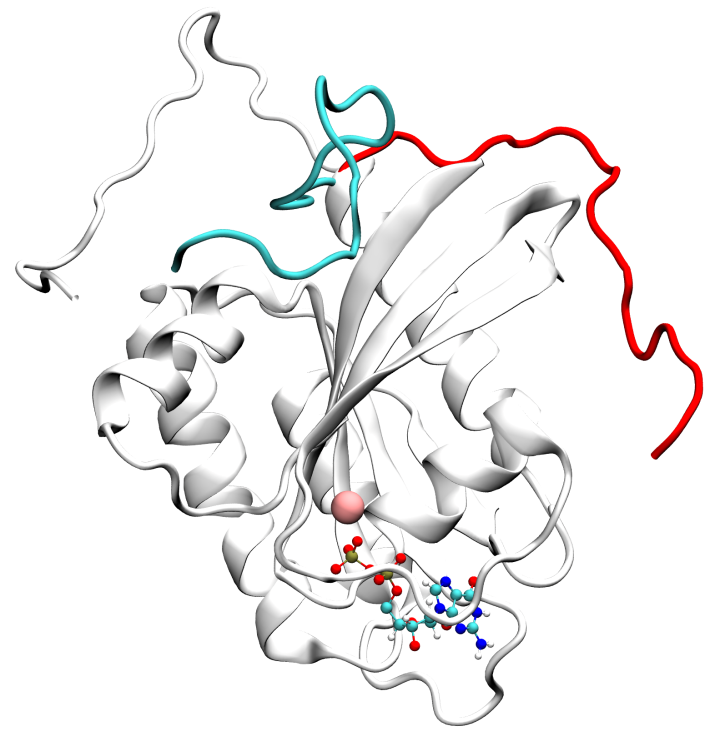


**Supplementary Figure 1:** The three different HVR orientations of the K-Ras4B full length starting structures used in the MDeNM and MD simulations. The HVR is colored white for model1, cyan for model2, and red for model3. The nucleotide is represented as CPK, Mg^2+^ as pink VDW sphere.


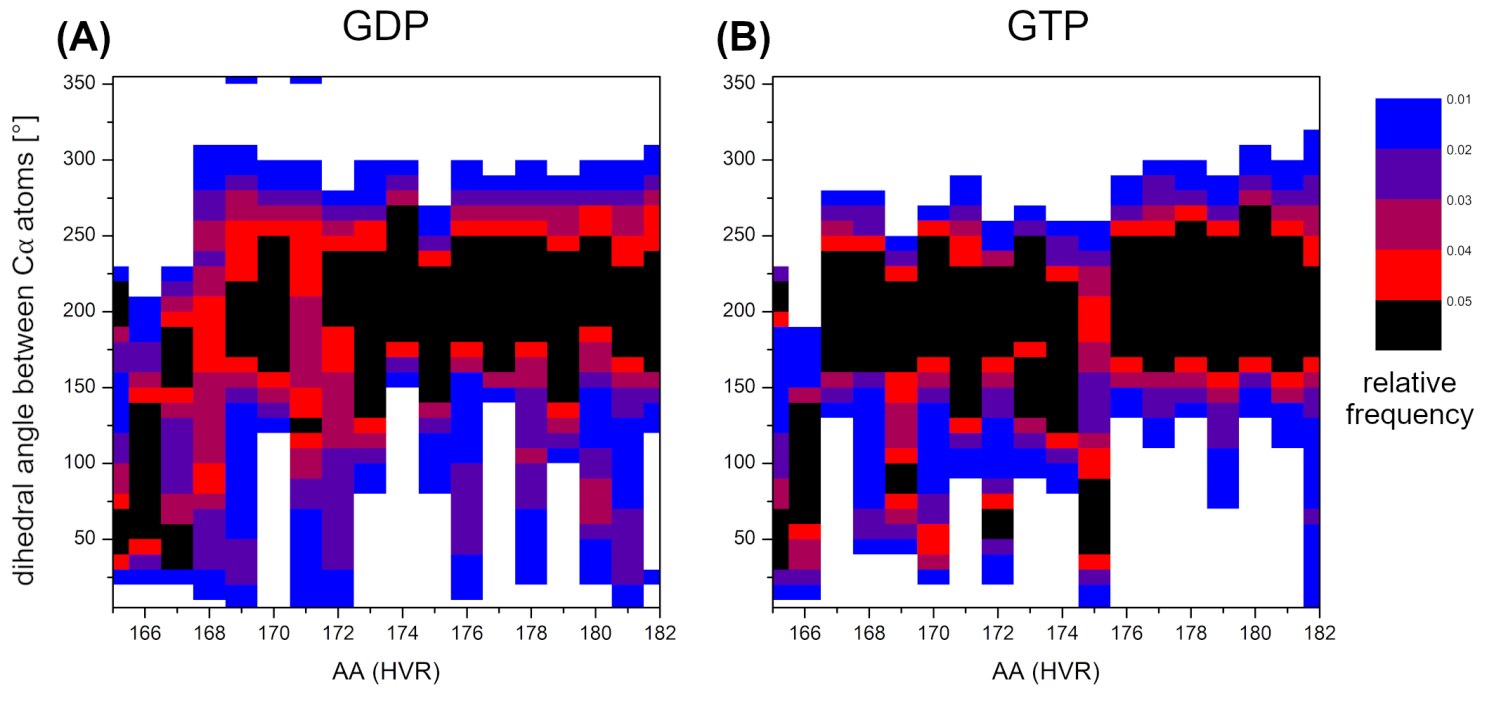


**Supplementary Figure 2:** Distribution of the dihedral angles defined by consecutive Cα atom quadruplets within the HVR in the **(A)** GDP-bound and **(B)** GTP-bound population. The first residue of each quadruplet is indicated by the abscissa, the dihedral angle by the ordinate, and the color-code corresponds to the populational relative frequency.


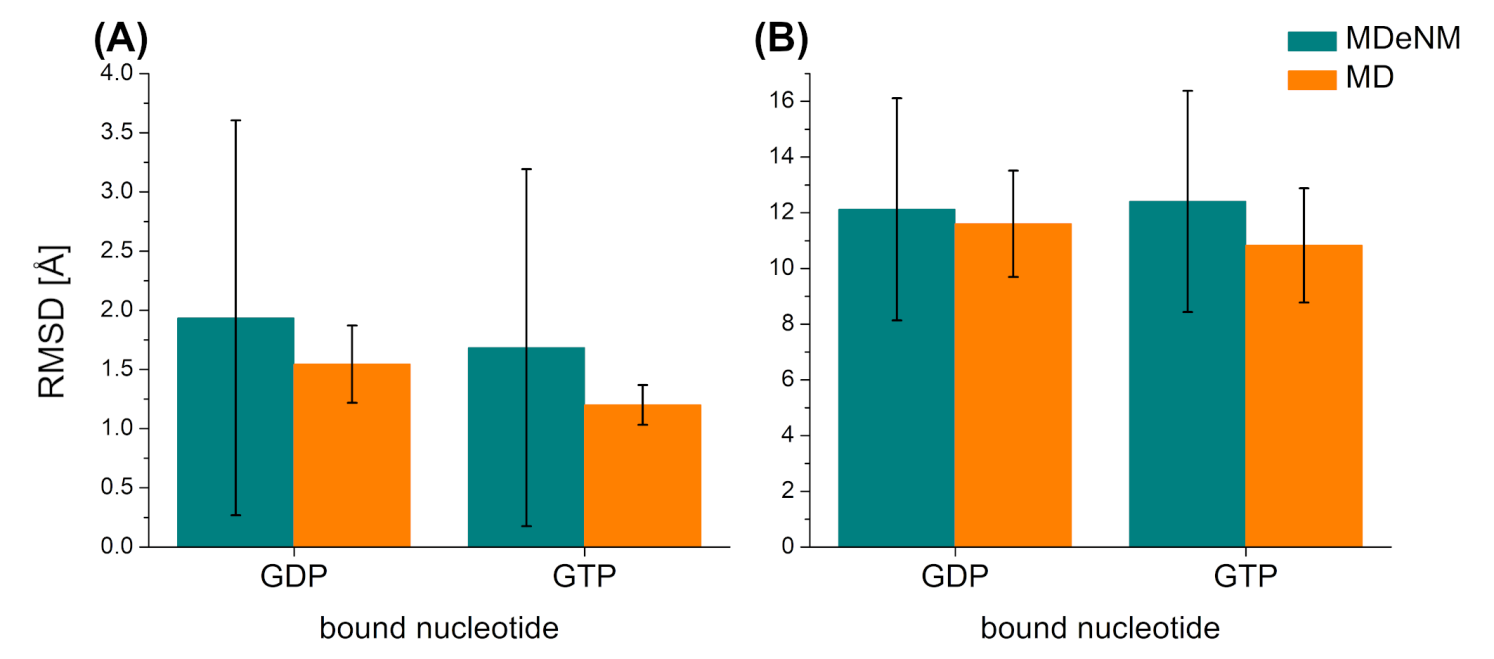


**Supplementary Figure 3:** Distribution of the RMSD for **(A)** the truncated (residue 1-166) and **(B)** full-length protein (residue 1-185) in the case of the MDeNM (dark cyan) and MD (orange) population for both activation states.
